# Non-Anatomical Surgical Solutions for Difficult Non-Unions: Case Series

**DOI:** 10.5812/traumamon.8563

**Published:** 2013-01-15

**Authors:** Galal Zaki Said, Osama Ahmed Farouk, Hatem Galal Said, Mohamed Mostafa Mohamed El-Sharkawi

**Affiliations:** 1 Department of Orthopedic and Trauma Surgery, Faculty of Medicine, Assiut University Hospitals, Assiut, Egypt

**Keywords:** Non-unions, Fracture, Non-anatomical, Forearm, Hip Fractures

## Abstract

**Abstract:**

Non-union occurs when bone healing ceases and does not continue without some type of intervention. Classification of non-union is traditionally based on the amount of callus or bone healing at the fracture site. Successful treatment of non-union often depends on appropriate reduction and realignment of the fracture, bone grafting if necessary, and stabilization. This may not be possible in some neglected and complicated non-unions. Under these circumstances, modification of the standard techniques or a limited surgical interference, that might not be anatomical, may succeed in improving function. We present four cases of non-anatomical salvage solutions for difficult long bone non-unions with satisfactory functional outcome.

## 1. Introduction

Non-union is defined chronologically as a fracture that does not unite in six months, or biologically when the healing process is lingering behind the expected rate (1). A defect in healing can be considered primarily biological, biomechanical, or mechanical. Classification of non-union is traditionally based on the amount of callus or bone healing at the fracture site. The Weber-Cech classification is widely applied (2). Hypertrophic non-unions show prolific callus formation. The environment is vascular and the healing potential is excellent, so inadequate immobilization or stabilization usually creates the non-union (3). Atrophic non-unions are characterized by absence of callus formation. The bone ends may be sclerotic or osteopenic. The healing environment is avascular. The fracture will not heal without changes to promote vascularity, such as introduction of living cells (autograft and/or free or rotational tissue transfer), removal of infection, and/or resection of nonviable bone (4). Successful treatment of a non-union often depends on appropriate reduction and stabilization of the fracture, in addition to bone grafting if necessary (5). In some neglected and complicated non-unions, anatomical open reduction and internal fixation techniques are not applicable. This may be due to previous repeated surgery, extensive bone loss, scarring of the surrounding muscles and unhealthy adherent skin. Under these circumstances, modification of the standard techniques or a limited surgical interference, that need not be anatomical, may succeed in improving function. The aim of this paper is to present non-anatomical surgical solutions for problematic cases of non-union of long bone fractures.

## 2. Case I

**Figure 1:**
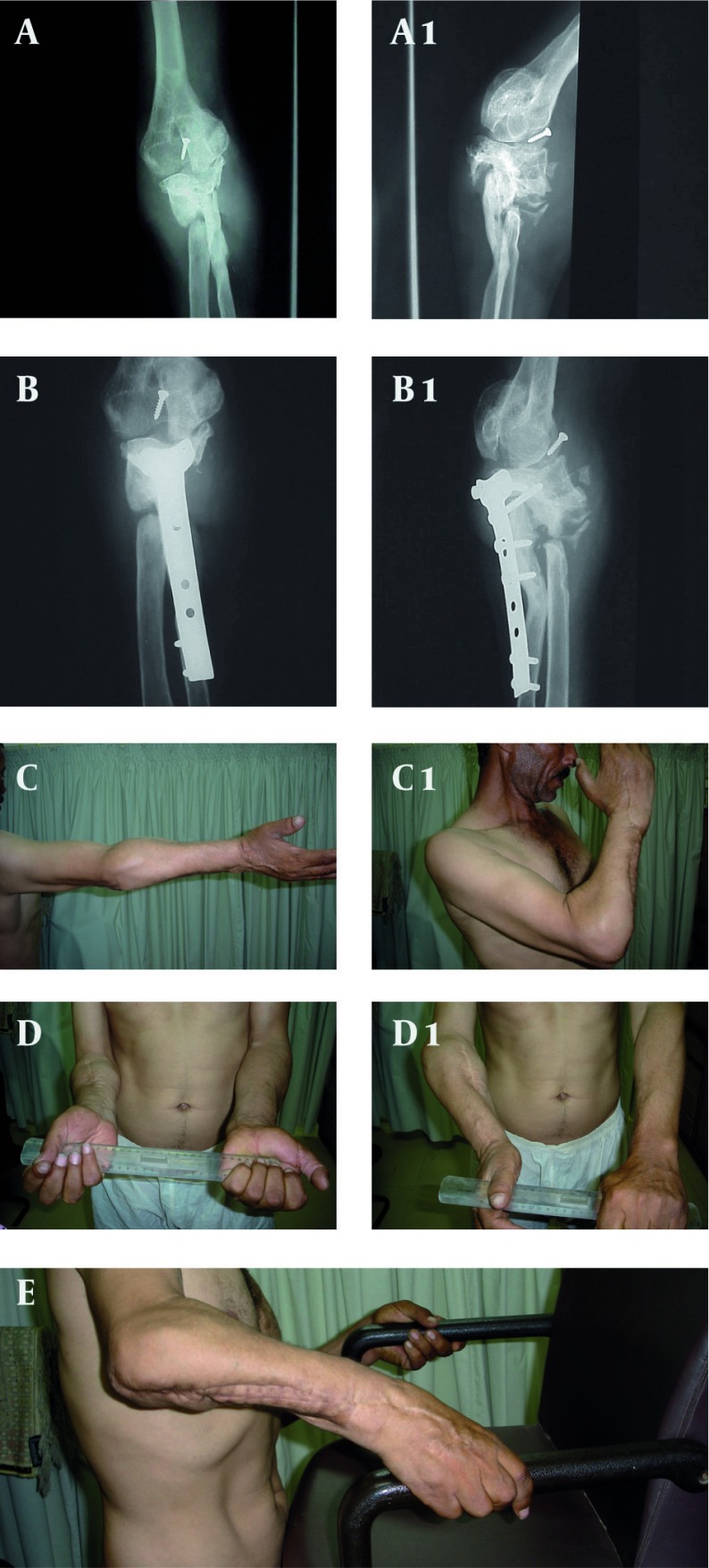
A, A1: Side Swipe Injury of the Right Elbow Four Years Previously Resulted in Non-union of the Upper Third of the Radius, Ulna. The Olecranon Fracture is nonunited. AP and Lateral Views of the Elbow and Forearm Showing the Upper Segments of the two Bones Fused Together in one Bony Mass. There is a Left-over Screw From Previous Surgery in Front of the Elbow Joint. B, B1: Three and Half Month Follow-up After T-plate Fixation of the Upper Bony Mass and the Distal Ulnar Shaft Showing Union. C, C1: Elbow Flexion Range 0-120°. D, D1: Full Supination, 90° Pronation at Last Follow-up. E: Patient Lifting a Chair.

A 55 year-old man travelling in a bus sustained sideswipe injury of his right elbow. This resulted in open comminuted fractures of the upper end of his forearm bones. After having multiple surgeries at the local hospital he presented four years later with a freely mobile non-union of the upper end of the radius and ulna, and was unable to use this limb. He had still useful range of movement at the elbow joint (20-90˚ flexion range). His DASH disability score was 54. (6) Radiological examination revealed that the upper ends of the radius and ulna to be fused together into one bony mass, with non-union between the bony mass and the remaining part of the shafts of the radius and ulna. The olecranon was nonunited, raised and ankylosed to the olecranon fossa (Figure1).

### 2.1. Surgical Procedure and Outcome

Surgery was limited to fixing the distal ulnar shaft to the proximal bony mass by a T-plate, after excision of the intervening scar tissue and freshening the bone ends. No bone graft or substitute was used. The ulna showed radiological union to the proximal bony mass at 14 weeks follow-up. At the last follow-up three years later, the patient had 10-120° elbow flexion range, full supination and 90° pronation occurring at the radial non-union site. His DASH disability score was 7.5 and he was able to use his arm as an agricultural laborer.

## 3. Case II

**Figure 2:**
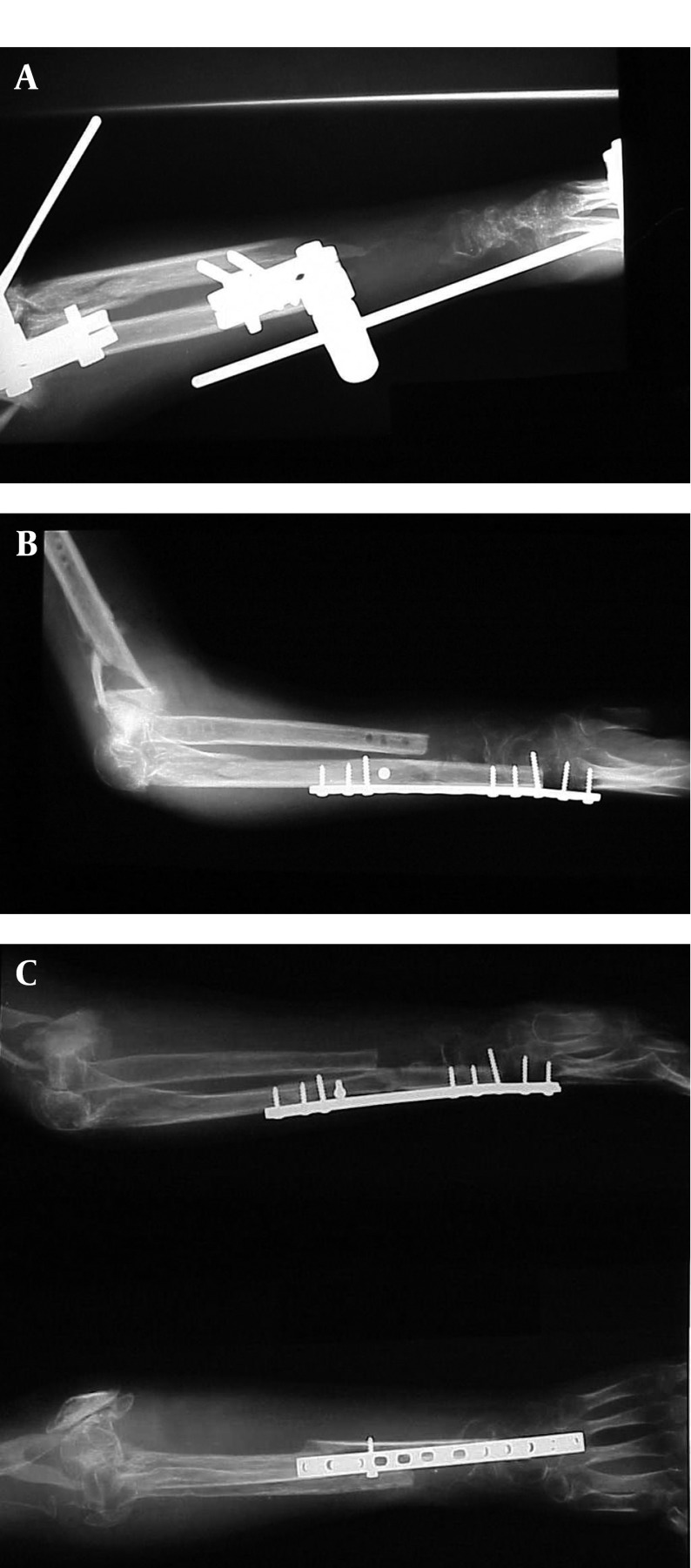
A. Motor Car Accident Resulted in Extensive Damage to the Left Elbow and Forearm. Bone Loss of the Lower Third of the Left Radius and Ulna, Leaving 2 cmMetaphyseal Segment of the Radius, on External Fixator at Presentation After Several Reconstructive Operations. B. Long Fibular Graft Between the Remaining Shaft of Ulna and the Metaphyseal Segment of the Radius Extending into the Carpus With Plate Fixation Resulted in one Bone Forearm. Three Months Postoperatively Showing Union. C. Six Month Follow-up, the Patient had to Change his Job to a Clerical Worker.

A 30 years old man sustained a motorcar accident, which resulted in Type III open fractures of the distal part of the left humerus and forearm bones. He had six operations of debridement, skin coverage, external fixation and sequestrectomy. His radiographs showed disorganized elbow, and loss of considerable part of the distal radius and ulna leaving 2 cm metaphyseal segment of the radius, (Figure 2). He had useful hand function and elbow flexion range 0-90°, yet he was unable to use his upper limb.

Months Postoperatively Showing Union. C. Six Month Follow-up, the Patient had to Change his Job to a Clerical Worker.

### 3.1. Surgical Procedure and Outcome

As a treatment of the non-union and bone loss of the forearm, a fibular graft was inserted between the remaining proximal segment of the ulna and the residual segment of the distal radius extending into the carpus. A long plate provided stabilization of the construction. After 12 weeks the graft has united at both ends and a one-bone forearm with arthrodesis of the wrist was produced. After one year he was satisfied with his limb function with DASH disability score of 8.3, but he had to change his job to a clerical worker.

## 4. Case III

**Figure 3:**
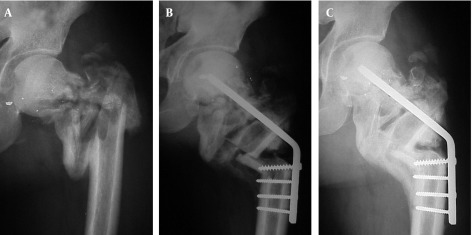
A. Bullet Injury Resulting in SevereComminution of Upper Left Femur and Coxa Vara. B. Post-operative X-ray After Wedge Removing Valgus Osteotomy Below the Non-union Mass and 130° Plate Fixation. C. Follow-up, Five Months Later Showing a Solid Bony Mass Uniting the Femoral Neck Fracture and the Osteotomy.

A man aged 27 years had a bullet injury of his left hip that resulted in shattering of the upper end of the femur. He presented four months later with nonunited fracture of the femoral neck, severe comminution of the trochanteric region, coxa vara, external rotation deformity and 5 cm. shortening (Figure 3).

Mass and 130° Plate Fixation. C. Follow-up, Five Months Later Showing a Solid Bony Mass Uniting the Femoral Neck Fracture and the Osteotomy.

### 4.1. Surgical Procedure and Outcome

A 130° angled plate was introduced along the axis of head and neck of femur and wedge removing osteotomy below the bone mass was performed after correction of the rotation deformity. The femoral shaft was pulled laterally and distally to reach the plate and to close the wedge and then fixation was completed. The neck shaft angle was restored, external rotation deformity was corrected and there was a regain in length of 4 cm. Union of the femoral neck fracture and the osteotomy site was achieved after 12 weeks. At three years follow-up he was walking with a minor limp and was back to his job as a construction worker. His Harris hip score improved from 38 to 89.

## 5. Case IV

**Figure 4:**
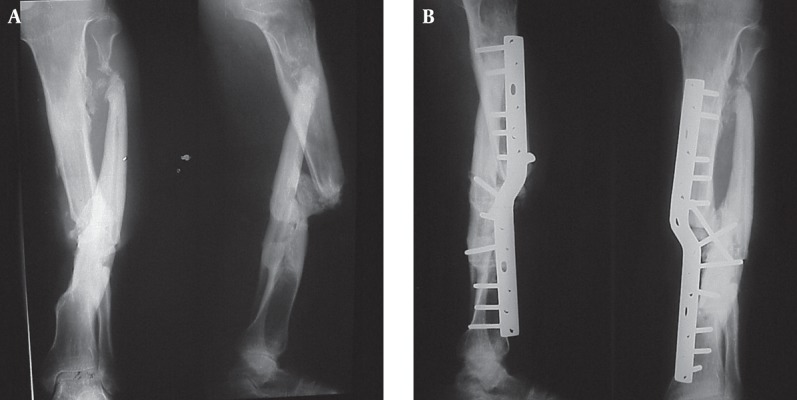
A.The Lower Tibial Fracture is United in Angulation. There is Callus Formation at the Tip of Nonunited Upper Tibial Segment. B. Union Three Months After Osteotomy at the Callus Site and Fixation by Bayonet-shaped Bent Plate

A 47 year-old man presented with freely mobile non-union of the left tibia and fibula on which the patient was walking for many years. With every step the leg angulated excessively on weight bearing and straightened back again. X-ray showed segmental fractures of the tibia and fibula (Figure 4). The central segment of the tibia had malunion at the distal fracture site, but failed to unite at the proximal one. Instead, it had united to the central segment of the fibula, which showed hypertrophy. The proximal fibular fracture was non-united.

### 5.1. Surgical Procedure and Outcome

At surgery there was excessive scarring with adventitious bursa and callus bone formation at the site of tibial pseudarthrosis. Axial alignment of the proximal and distal segments of the tibia was achieved through osteotomy of the callus mass at the tip of the proximal segment. This left the central tibial segment nonaligned because of the lower malunion. A broad DCP was contoured in a bayonet shape and used for fixation. Two lag screws securely compressed the fragments. Partial correction of the shortening was obtained through straightening of the leg. After 12 weeks bony union was achieved and he was allowed full weight bearing, with a heel lift to compensate 2 cm. of shortening. Two years later, he was satisfied with the result of treatment and was fully active in heavy work.

## 6. Discussion

Anatomical open reduction and internal fixation of neglected or complicated mal- and non-unions are sometimes not possible. Many of these patients have had several surgeries before, resulting in scarring of the surrounding muscles and adherent unhealthy skin. Under such circumstances, the minimum surgical interference addressing the basic principles of correction of axial and rotational deformities, and correction of as much as possible of the lower limb shortening, are the recommended procedures.

Case I. Sideswipe injuries of the elbow are devastating injuries that often result in serious disability and loss of function (7, 8). Kinzel et al. reported on 10 cases of side swipe injuries of the elbow that were treated by primary open reduction and internal fixation of the humerus, radius and ulna, nine of them needed additional surgical procedures. Eight patients had extensive functional deficit (9).

In our patient, the proximal segments of the radius and ulna were fused in one bony mass. It would have been unlikely to get supination-pronation movement, if a trial to resolve this bony mass and anatomical reconstruction would have been made. Uniting the ulnar non-union provided stability to the forearm. Allowing supination-pronation to occur at the radial pseudarthrosis and flexion-extension at the olecranon pseudarthrosis site was successful salvage treatment in this patient.

Case II. One bone forearm is an old solution for extensive loss of bone segment of the radius (10-12). This could be achieved by inserting the ulna into the metaphyseal segment of the radius. In our patient with bone loss of both forearm bones, a fibular graft between proximal ulna and distal radius can achieve the same aim. This resulted in a stable forearm with good functional and cosmetic results as well as patient satisfaction.

Case III. The fragments of the upper femoral fracture were left untouched in varus position. Valgus osteotomy below the bone mass and internal fixation by single-angled blade-plate succeeded in achieving union of femoral neck fracture with correction of the varus deformity and length. It also tensioned the abductor muscles. Subtrochanteric valgus osteotomy is a classic procedure to correct post-traumatic deformities of the hip, without the need to undo the healed comminuted fracture and attempt open reduction. The traditional method of fixing the osteotomy by double angled plate has the drawbacks of medialisation and verticalisation of the femoral shaft, which results in valgus strain of the knee joint. On the other hand, single-angled blade-plate allows pulling the femoral shaft laterally and distally to the plate, along the slope of the obliquely situated osteotomy line. This results in correction of medialisation of the femoral shaft, restoration of the normal inclination of the femur in the coronal plane and correction of shortening (13).

Case IV. For segmental diaphyseal fractures in which one fracture site has malunited and the other did not, attempt at anatomical reduction of the malunion is anticipated to result in stripping of the central bone segment of its soft tissue attachments. This is undesirable especially so if the surrounding muscles are scarred (14). A long plate fixing the tibial non-union and aligning the proximal and distal segments of bone, and leaving the central segment non-aligned was a practical solution in this patient.

## 7. Conclusions

Restoration of the anatomy should always be the aim in treating nonunions. However, in some unusual non-unions, a salvage surgical interference that might not be anatomical may succeed in improving the function. The authors are presenting their own experience in treating four exceptional cases.
